# Chronic subdural haematoma: a case series of uncommon presentations of a common disease

**DOI:** 10.1093/jscr/rjad649

**Published:** 2023-12-06

**Authors:** Uchenna Ajoku, Linda Iroegbu-Emeruem

**Affiliations:** Department of Surgery, University of Port Harcourt Teaching Hospital, Port Harcourt, Rivers, Nigeria; Department of Surgery, Rivers State University/ Rivers State University Teaching Hospital, Port Harcourt, Rivers, Nigeria

**Keywords:** chronic subdural haematoma, computerized tomography, oculomotor palsy, paraplegia, foot drop, roller coaster

## Abstract

Chronic subdural haematoma is a common neurological condition especially among the elderly population. Its presentation can be unspecific but often attributed to pressure, cortical irritation, and/or vascular compromise. In the patients’ cohort presented below, we have a series of cases where clinical history and examination did not result in an initial clinical diagnosis or suspicion of chronic subdural haematoma, with the diagnosis made only after brain imaging. We reviewed the literature regarding the aetiopathogenesis and clinical features of our patient cohort, which included a young woman with isolated right ptosis and pupillary dilatation, an elderly man with paraplegia, another elderly man with isolated right foot drop, and a young military man with no history of trauma. Our series re-emphasizes the sometimes non-specific and varied clinical presentation of chronic subdural haematoma. We reiterate the need for early brain imaging in patients who present with neurological disorder.

## Introduction

Chronic subdural haematoma (CSDH) is a common neurological condition especially among the elderly population [1–3]. The aetiology and pathogenesis of CSDH are multifactorial. Although, various pathologic processes may be at play in its development in different clinical scenarios, it is likely that these processes occur concurrently in the evolution of the disease. Trauma is the leading cause in majority of cases. In ~50% of the time, there may not be a history of a traumatic event [2].

The initial trauma may be so mild, especially in the elderly, that they may go unrecognized. The haematoma that forms may progress insidiously over a period during which the patient may be asymptomatic. With the initial formation of acute SDH, an inflammatory process begins which leads to the development of a fibrous membrane along the internal and external layers of the clot. With the passage of time, this new membrane undergoes reorganization several weeks after injury [4].

Inflammatory response results in the expansion of the haematoma and perpetuation of the pathological process [5, 6]. This response involves the overproduction of proinflammatory mediators such as vascular endothelial growth factor, interleukin (IL)-6, IL-8, tumour necrosis factor-α (TNF-α), matrix metalloproteinases, and basic fibroblast growth factor [7–9].

In addition to the inflammatory response, abnormal angiogenesis contributes to increased clot volume through persistent micro-haemorrhages and rebleeding, thus creating a vicious cycle of inflammation and haemorrhage. Activation of the coagulation mechanism and attendant hyperfibrinolysis results in a local coagulopathy within the haematoma cavity. This has been demonstrated by Shim *et al.* and Kawakami *et al.* [10, 11].

The symptom-complex of CSDH is either from direct physical compression of an anatomic area of the brain or an indirect effect from compromise of a particular vascular territory [12, 13]. The clinical presentations of CSDH are sometimes non-specific. Symptoms and signs are often attributed to intracranial hypertension, direct cortical irritation/compression, and vascular compromise [14]. In the patients’ cohort presented below, we have a series of cases where clinical history and examination did not help in the clinical diagnosis or suspicion of CSDH, the diagnoses were made only after brain imaging. This re-emphasizes the need for early brain imaging in patients with suspected brain pathology.

## Case description

### Case 1 – unilateral ptosis

**Figure 1:**
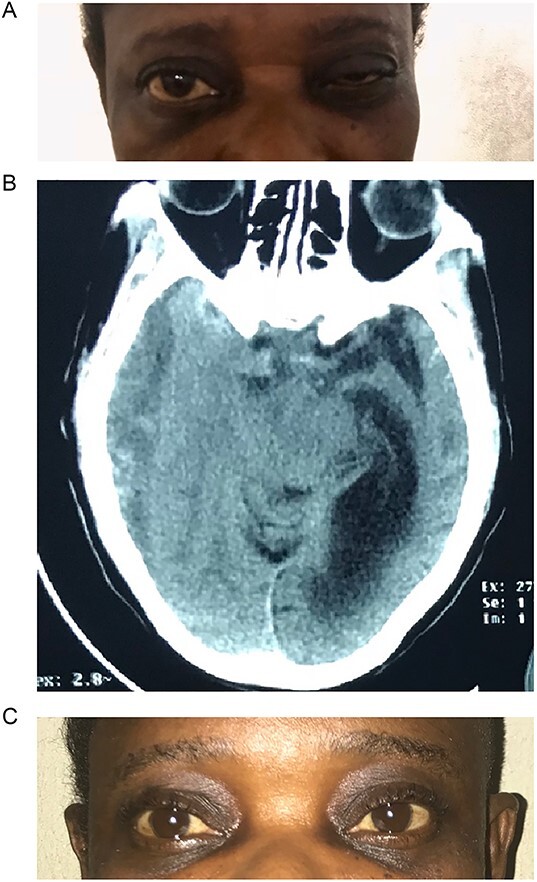
(A) Pre-op with right ptosis/ophthalmoplegia. (B) Pre-op axial CT showing right CSDH. (C) Post op with resolution of ptosis.

A 42-year-old woman presented to the outpatient clinic with a 2-week history of headaches and 3-day history of drooping of the right eye and inability to open the eye. She had been involved in a passenger road traffic accident 3 weeks prior. At presentation she was fully conscious, and oriented in time, place, and person. However, she had a recent onset painless drooping of the right eyelid (Fig. 1A). Her vision was preserved with dilated pupils that reacted very slowly to light as well ophthalmoplegia. Other cranial nerves were normal on examination. There were no long tract signs.

Brain CT scan done revealed a right fronto-temporal CSDH causing uncal compression and midline shift as well as partial effacement of the ipsilateral lateral ventricle (Fig. 1B).

She underwent a right frontal and parietal burr hole drainage of the haematoma under local anaesthesia and sedation. Her post-operative course was satisfactory, and she was discharged on the 4th day post-surgery. She had complete resolution of her symptoms in 2 weeks (Fig. 1C).

### Case 2 – paraplegia

A 72-year-old man who was admitted by the orthopaedic team on account of progressive lower extremity weakness of 3 weeks duration that culminated in his inability to ambulate independently. He had no other neurologic symptoms. He denied history of prior trauma. Although he was a known hypertensive, he had not been on antiplatelets. His neurologic examination revealed a fully conscious elderly man who was awake, alert, well oriented in time, place, and person. Other neurologic examination findings were normal except for grade 2 power in both lower limbs. His deep tendon reflexes were normal in all extremities. Sensory function was intact.

**Figure 2:**
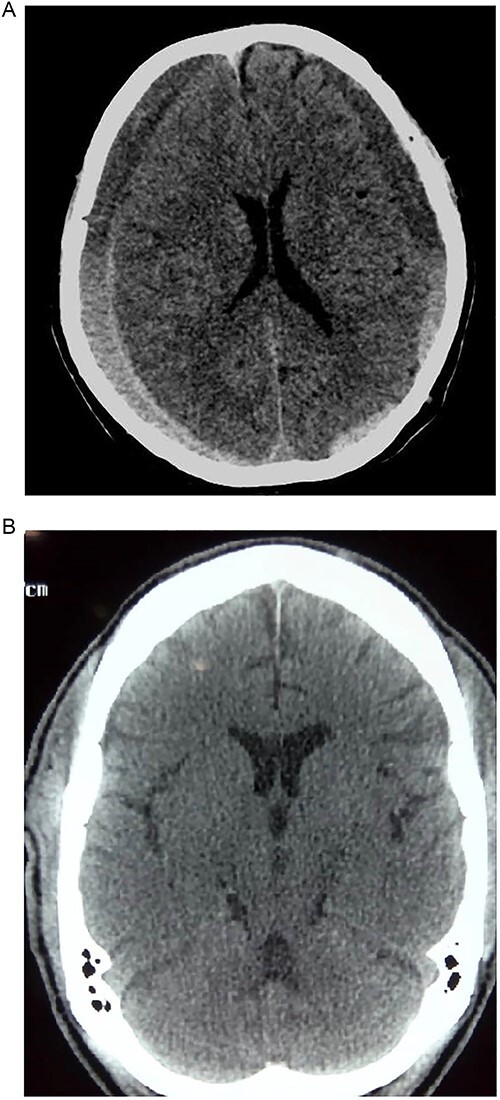
(A) Pre-op showing bilateral CSDH. (B) Post op complete resolution of CSDH.

Prior to this, he had been managed for low back pain and lumbar spondylosis. Spine MRI done prior to neurosurgery consultation did not explain the lower extremity motor weakness. Brain CT done revealed bilateral fronto-parietal CSDH (Fig. 2A). He was worked up for urgent surgical evacuation through a bilateral frontal and parietal burr hole drainage. He made good neurologic improvement and his lower extremity power returned to normal on the 8th day post op. Post-operative CT scan done at 4 weeks follow-up showed satisfactory resolution of the haematoma (Fig. 2B).

### Case 3 – left isolated foot drop

**Figure 3:**
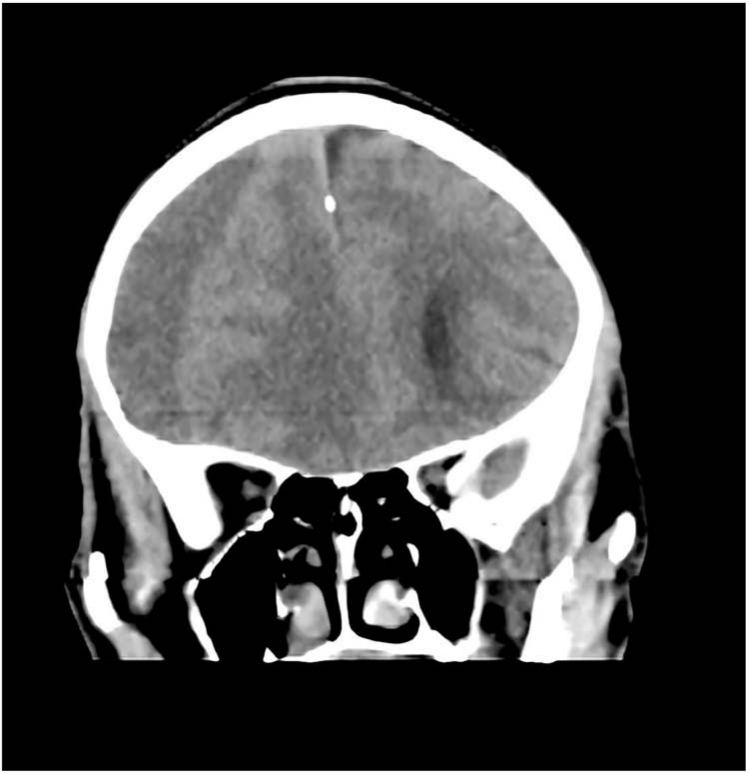
Coronal CT with right CSDH.

**Figure 4:**
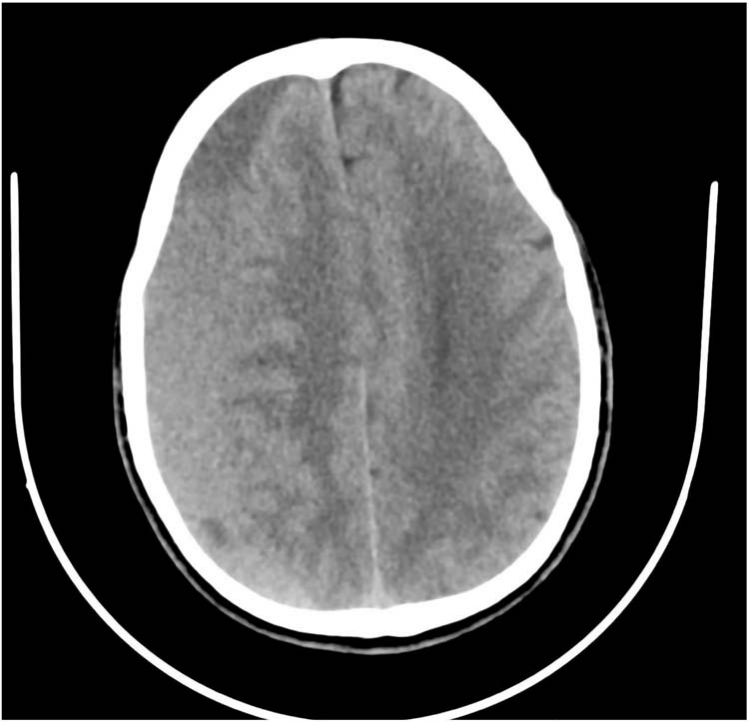
Axial CT showing CSDH.

A 78-year-old man with a 1-week history of headaches and dragging of the left foot. He had fallen in the bathroom 6 weeks prior to presentation. He had no other symptoms. Examination was unremarkable except for a left-sided foot drop with increased ankle jerk reflex. Brain CT done revealed a right-sided fronto-parieto-temporal CSDH (Figs 3 and 4). He had burr hole drainage and was discharged 3 days later to continue physiotherapy at home. At 4 weeks follow-up, his symptoms had completely resolved.

### Case 4 – spontaneous CSDH in a young fit adult following ‘Bumpy Rides’

**Figure 5:**
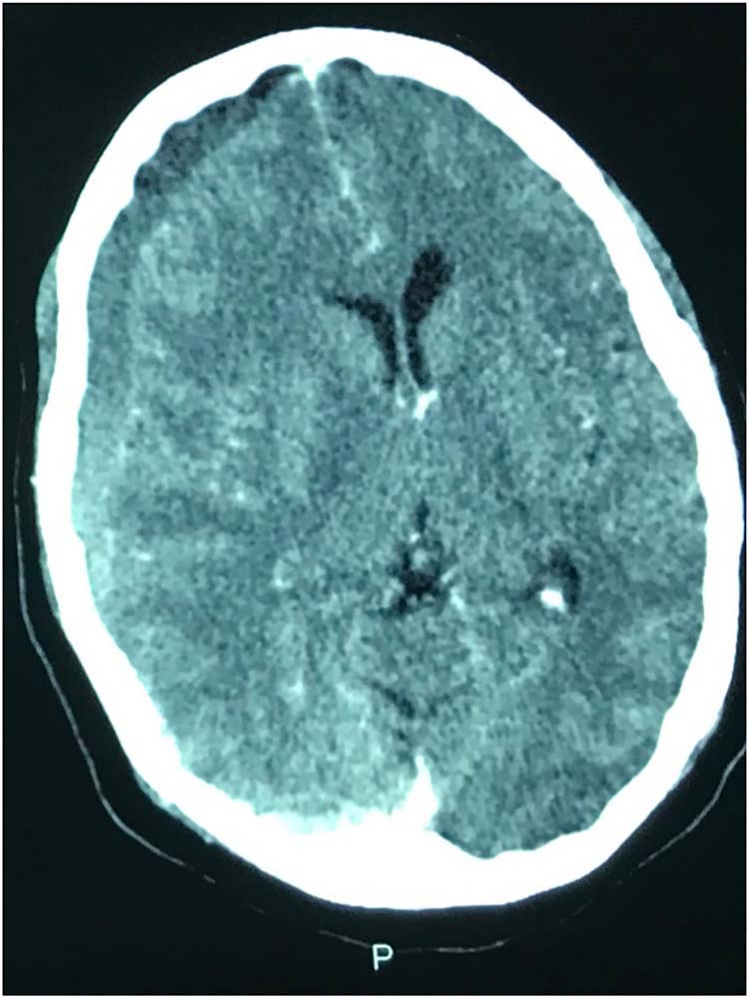
Axial contrast CT showing right CSDH.

A 40-year-old military man who presented with a 1-week history of severe generalized headaches that was unresponsive to analgesics. He had two episodes of non-projectile vomiting before presentation. There were no other symptoms. He denied any history of trauma. He was not on any anticoagulants and had been otherwise healthy. Neurologic exam was normal. Brain CT scan done showed a right-sided fronto-parietal CSDH (Fig. 5). With this finding, he was further questioned about his daily routines/activities. It was at this point that he admitted to being part of an oil pipeline surveillance team in the Niger Delta region of Nigeria. He recounted that his job involved riding in an open van on very bumpy roads. And so, given the absence of any trauma, we hypothesized that this ‘roller coastertype phenomenon may be responsible for his CSDH. He underwent burr hole drainage and had complete resolution of his symptoms in 48 hours and was discharged home on the 3rd day post op.

## Discussion

Symptoms and signs of CSDH are notoriously unspecific. This has led to CSDH being reported in neurological literature as a ‘great mimic of neurological disorders’ [15]. Clinical presentation includes but not limited to cognitive impairment, long tract symptoms and signs, and cranial nerve palsies. Although ophthalmological manifestations of CSDH are uncommon, when present, they may include papilledema, visual blurring, and visual field defects.

Isolated third nerve palsy is extremely rare, with only ~16 cases reported in literature [16]. Of the 16 cases reported, one was associated with bilateral CNIII palsy after evacuation of CSDH. This was found to have resulted from pontine haemorrhage after the haematoma evacuation. Eleven of the 16 cases reported in literature had full recovery following surgery, 4 had partial recovery, and 1 did not recover. From a pathophysiologic standpoint, it is caused by direct compression of the right oculomotor nerve by the medial temporal lobe. During initial clinical evaluation, our patient was suspected of having right posterior communicating artery aneurysm. However, brain CT revealed a massive right-sided CSDH with uncal herniation and complete effacement of the basal cisterns (Fig. 1C). The oculomotor nerve was likely compressed by the uncus against the tentorial edge. The nerve is a mixed somatic and visceral motor nerve which innervates the levator palpebrae superioris and the extraocular muscles except the lateral rectus and the superior oblique muscles. It also carries parasympathetic fibres to the pupillary constrictors and the annular portion of the ciliary muscle. Pressure on the oculomotor nerve can cause paralysis of the levator palpebrae muscle (ptosis), pupils that are non-reactive to light and restricted eye movement as described in our patient.

Another unusual presentation was paraparesis in a 72-year-old man. With a long-standing history of low back pain and lumbar spondylosis, an initial diagnosis of degenerative spine disease was considered. However, with an unremarkable spine MRI, a brain CT was done and it revealed a bilateral CSDH with extension into the anterior inter-hemispheric fissure. We postulated that the bilateral motor involvement of the lower extremity could be from two possibilities.

The first possibility was a direct compression of the lower limb motor strip against the falx cerebri from the bilateral subdural collection like what is seen in a parasagittal meningioma [13].

Second, subfalcine herniation could result in the compression of the branches of the anterior cerebral artery with resultant ischaemia of the medial surfaces of the frontal lobes, which bears the motor homunculus of the lower extremities. Koyama *et al.* [13] demonstrated a vascular aetiology in case of interhemispheric SDH causing paraparesis. In their patient who had left hemiparesis and right interhemispheric SDH, an angiography demonstrated a lateral displacement of the right callosomarginal artery and an avascular area between the falx and the callosomarginal artery. Our patient made a dramatic clinical improvement once the haematoma was drained and was functionally independent within 1 week after surgery.

While few reports of bilateral lower extremity weakness have been reported with CSDH, unilateral foot drop is extremely rare. Two previous cases of isolated foot drop from CSDH have been reported, Sengupta *et al.* [17] and Weisberg [18]. Direct cortical compression of the motor foot area of the homunculus against the falx cerebri is the likely explanation in this scenario given the large right hemispheric CSDH. While the clinical suspicion of an intracranial lesion was considered due to raised ankle reflex, a diagnosis of CSDH before imaging was not entertained because of the rarity of isolated foot weakness.

In another unusual case, we review a rare case of a ‘roller-coaster’ phenomenon presenting with CSDH. Interestingly, extensive literature search did not yield any report of CSDH occurring with bumpy road rides in a young fit adult. Instead, few reports of CSDH occurring from acceleration-deceleration-type phenomenon have been reported in literature.

Scranton and colleagues described a case of symptomatic bilateral SDH associated with riding a centrifugal motion simulator in a previously healthy 55-year-old male [19]. The suspected mechanism for this injury was rotational acceleration-deceleration forces acting on the brain parenchyma and intracranial vasculature. This theory was hypothesized by Gennarelli and Ommaya following their works in primate models. Bumpy rides such as those experienced by our patient produces many vector forces with linear, angular, and rotational components and could potentially cause vascular injuries [20].

Williams *et al.* reported roller coaster headaches and subacute SDH in an otherwise healthy 45-year-old female with no other medical problems [21]. The patient reported riding a roller coaster 5 weeks prior, after which she developed moderate occipital headache, which worsened over time. She had drainage of the SDH and made full neurologic recovery.

Rotational acceleration-deceleration forces have been found to cause very rapid deformations in the brain parenchyma as well as brain vasculature. These forces are measured in G, where 1G is the acceleration experienced by a free-falling object near the surface of the earth or 9.81 m/s2 or 32.2 ft/s. The magnitude, rate of change, and duration of G forces at play are important determinants of injury [22, 23].

The report of CSDH following eccentric exercise using vibrating belts by Park *et al.* [24] supports the multidimensional nature of these G forces. It is most probable that lateral G forces, rotational acceleration, abrupt directional changes, and predisposing anatomic factors like cerebral atrophy play important roles in the aetiology of these injuries.

The effect of these G forces alone does not tell the whole story. An important finding is that individual brain deformation differs significantly, and this may be the reason for the differences in injury susceptibility among different people [25]. Even though our patient was a healthy young military man, the acceleration-deceleration forces he was exposed to daily may have caused rupture of his bridging veins and resulted in subdural bleeding.

## Conclusions

This series of uncommon presentations of a common neurosurgical disease re-emphasizes the non-specific features of CSDH especially in the older population. All four of the patients did not have CSDH as their initial diagnosis. However, on imaging the diagnosis of CSDH was made, underscoring the value of routine early brain imaging in patients who present with florid neurological symptoms and signs such as the patient cohort in this series.

## Data Availability

Data sharing are available from the authors upon reasonable request and with the patient’s permission.
